# Supplementary material for: Thermal limits of bumblebees and honeybees are modulated by different functional traits: predictions of a mechanistic model
Source: PLoS One. 2025 May 6;20(5):e0320038. doi: 10.1371/journal.pone.0320038 (PMC12054886; doi:10.1371/journal.pone.0320038)
Supplement: S1 Appendix — (PDF) [file pone.0320038.s001.pdf]

Supporting Information

October 10, 2024

1 Modelling evaporative cooling

We use a model of evaporative cooling developed by [30]. This model takes the form $Q_{evap} = \dot{m}_{evap} h_{fg}$ where $\dot{m}_{evap}$ is the rate of evaporation (in kg/s) and h_{fg} (J/kg) is the latent heat of vaporization. The model we use assumes that the surface of the evaporating fluid is a hemisphere of radius R_0. Under this model,

$$\dot{m}_{evap} = 2\pi R_0 \rho D_A \ln\left(\frac{1 - Y_{air}}{1 - Y_{sfc}}\right) \tag{1}$$

where ρ is the density of air, D_A is the diffusion coefficient of air into humid air, Y_{air} is the mass fraction of of water vapor in the ambient air, and Y_{sfc} is the mass fraction of water vapor in the air at the surface of the evaporating fluid. The mass fraction of a substance in a mixture is the ratio of the mass of the substance to the total mass of the mixture. These mass fractions can be calculated based on the corresponding mole fractions (ratio of the moles of the substance to the total moles of the mixture), X_{air} and X_{sfc}, using the formula

$$Y = \frac{1}{1 + \frac{1-X}{X}\frac{MM_{air}}{MM_{vapor}}} \tag{2}$$

where MM_{air} is the molar mass of air and MM_{vapor} is the molar mass of vapor. The mole fraction is given by $X = \dfrac{p_v}{P}$, where $p_v = r_h p_{sat}(T)$ is the partial pressure of water vapor in humid air, calculated based on the relative humidity r_h and the saturation pressure at air temperature T, $p_{sat}(T)$. The saturation pressure is given by the Clausius-Clapyron relation, $p_{sat}(T) = Ae^{\frac{B}{T}}$ [30]. For the mole fraction X_{air}, we use the relative humidity of the ambient air and the ambient air temperature. For the mole fraction X_{sfc}, we use 100% relative humidity and the temperature of the nectar droplet, which we assume to be the head temperature for simplicity. Since the bee rapidly moves the droplet in and out of its mouth, the temperature should remain very close to the head temperature.

The model we use was developed for evaporative cooling of a coffee cup in still air, and uses the diffusion coefficient of dry air into humid air, $D_A = 2.06 \cdot 10^{-5}$ m^2/s. However, as the honeybee is flying at a speed of v m/s, we use the Peclet number $\dfrac{vL}{D}$, where L is the width of the droplet of nectar, to relate the diffusion process occurring in the evaporation to the advection process of the bee's flight. Setting the Peclet number equal to 1 for equivalent diffusion and advection processes, we have $D = vL$.

2 Nondimensionalization and Steady State Analysis

We nondimensionalize a simplified form of the model with no heat transfer between the head and thorax, and with no behavioural cooling. The nondimensionalized form is

$$\frac{dx}{dy} = K_1 \exp -K_2\left(\frac{1}{x} - 1\right) + K_3 + K_4 x + K_5 x^4 \tag{3}$$

Parameter	**2 steady states (low value)**	**4 steady states**	**2 steady states (high value)**
E	$0.009 \cdot 1.6022 \cdot 10^-19$ J	$0.63 \cdot 1.6022 \cdot 10^-19$ J	$0.63 \cdot 1.6022 \cdot 10^-19$ J
s	0.9965	0.9965	0.5
v	2.1 m/s	2.1 m/s	2.1 m/s
T_{air}	15°C	15°C	15°C

Table 1: Parameter values which can be changed from the default or fitted values to achieve each steady state condition.

with dimensionless variables $x = \frac{T_{th}}{T_i}$ and y $= \frac{t}{\tau}$ where T_i is the reference temperature for the metabolic rate (see tables 5 and 4) and $\tau = 1$. The dimensionless constants are

$$K_1 = \frac{\tau i_0 \left(\frac{m_b}{m_i}\right)^{3/4}}{T_i m_{th} c}$$
$$K_2 = \frac{E}{kT_i}$$
$$K_3 = \frac{\tau a_{th}}{cm_{th}} \left(\frac{\epsilon_a(\alpha_p P + \alpha_{np}(Pf + \delta T_{air}^6 + \sigma T_g^4))}{T_i} + \frac{T_{air}\kappa \left(\frac{v}{\nu}\right)^n c_d l_{th}^{n-1}}{T_i} \right)$$
$$K_4 = \frac{-\tau a_{th} s \left(\frac{v}{\nu}\right)^n c_d l_{th}^{n-1} \kappa}{m_{th} c}$$
$$K_5 = \frac{-\tau a_{th} T_i^3 \sigma \epsilon_e}{m_{th} c}$$

The nondimensional model has either two or four steady states, depending on the parameter values. One unstable steady state is always at $T_{th} < 0$. When this steady state is near or below absolute zero, we disregard it, as different physics are required at temperatures near absolute zero. One stable steady state is always at $T_{th} > 0$. Varying any of the dimensionless constants may result in a saddle node bifurcation (depending on the values of the other constants) to create an additional pair of steady states with values $T_{th} > 0$. One example of how this can occur is shown in figure 1. If there are two steady states, the stable steady state generally has either a value less than approximately 100°C, or a very high value of several thousand degrees or more. Parameter values that result in each of these conditions are provided in table 1. Many other combinations will also work. A mathematica notebook that can be used to produce these results is provided.

As this analysis is carried out with a simplified model, we are not concerned with the exact values of the dimensionless constants and steady states. Numerical solutions indicate that the general pattern holds for the full model. When solving the full model numerically, if the upper pair of steady states exists, it appears that fast dynamics near the initial condition and a large step size may occasionally put numerical solutions into the upper basin of attraction even for initial conditions in the lower basin of attraction.

3 Global Sensitivity Analysis

3.1 Method

In order to determine the sensitivity of the model output (equilibrium thorax temperature) to changes in parameter value, we allow solutions in which the thorax temperature is higher than the lethal limit. To reduce computation time and exclude any solutions which are incorrectly at the higher equilibrium value (see above), we halt any solutions that go above 500°C and remove these solutions from the sensitivity analysis.

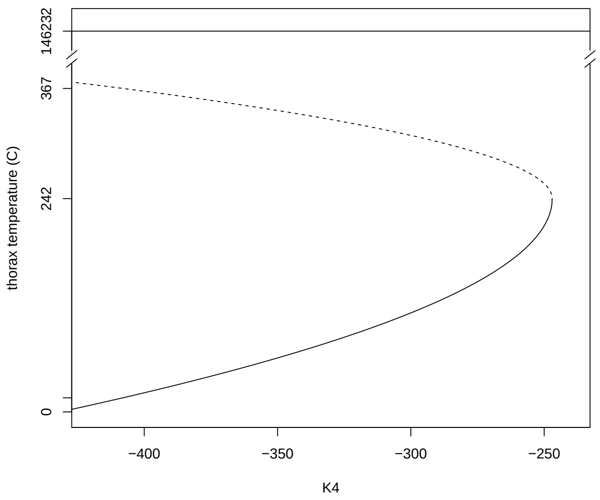

Figure 1: Sketch of a bifurcation diagram for K_4 showing the saddle node bifurcation. The remaining non-dimensional parameters are held at fixed values. The original parameter values are set as in column 2 of table 1, to produce the nondimensional parameters $K_1 = 0.0099$, $K_2 = 24.5219$, $K_3 = 396.135$, $K_4 = -408.447$, and $K_5 = 0.000716995$. We then vary K_4 between -200 and -410. Plot is not to scale.

We follow the method for a global sensitivity analysis outlined by [28]. Although this method was developed with stochastic models in mind, the authors recommend doing a single simulation per parameter sample, and therefore the method is applicable to a deterministic model for which it is only possible to have a single solution per parameter sample. The steps we follow to carry out the sensitivity analyses are as follows:

1. Create a set of parameter samples from the ranges provided for each parameter. We do this by taking a random latin hypercube sample with n samples of $k = 30$ values ($n = 10000$ for bumblebee; $n = 30000$ for honeybee, as determined in step 5) (using R package lhs [4]). We then shift and scale these values using the appropriate uniform or normal distribution for each of the model parameters so that each .

2. Solve the model for the equilibrium thorax temperature for each parameter sample.

3. Remove any solutions with equilibrium thorax values at or above $T_{th} = 100°$C.

4. Model the relationship of equilibrium temperature vs. each of the parameters using boosted regression trees as recommended by [28]. The boosted regression trees are fitted using the *gbm.step* function in the R package dismo 1.1-4 [14]. We use the recommended values for learning rate (0.01), bag fraction (0.75), tree complexity (3), and cross-validation (10-fold), and assume a gaussian distribution for equilibrium thorax temperature.

5. Check whether the sample size is sufficient by creating subsamples and using the measure of stability: $D_\beta = \exp\left(\sum_{j=1}^{2}\sum_{i=1}^{s}\frac{p_{ij}\ln(p_{ij})}{2} - \sum_{i=1}^{s} p_i \ln(p_i)\right)$, where j indicates the subsample, i indicates the model parameter, s is the total number of parameters, p_{ij} is the influence of parameter i calculated from subsample j, and p_i is the average of p_{i1} and p_{i2}. The value of D_β should approach 1, indicating that the influence of the parameters approaches equality between subsamples. If the sample size is not sufficient, additional samples can be added to the original set without affecting the latin hypercube distribution. (See figure 2.)

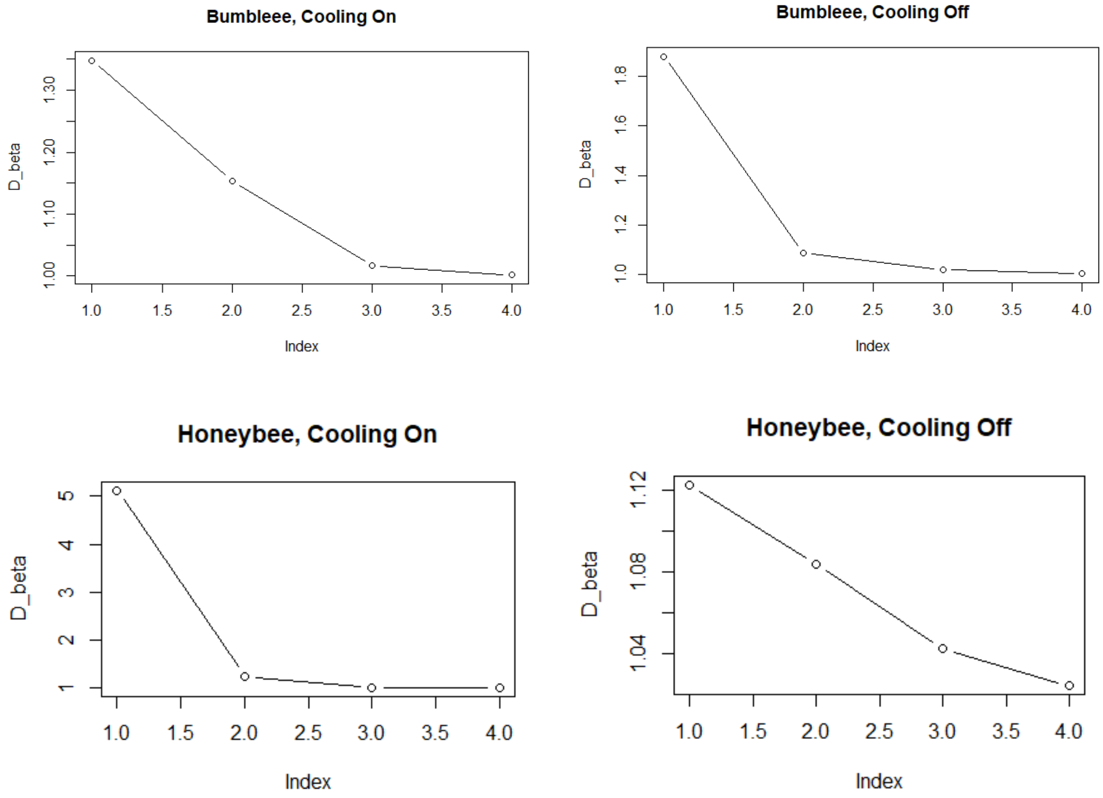

Figure 2: Stability D_β approaches 1 as parameter sample size increases, indicating a sufficient sample size in all cases.

3.2 Results

The relative influence of each parameter value is shown in table 2. For both honeybees and bumblebees, the air temperature, T_{air} is highly influential. We therefore present the main results of our paper in terms of varying air temperature. The flight speed, v is also highly influential, especially for honeybees. The metabolic rate, i_0 is also influential, and we know this varies naturally between resting and flying behaviours. We therefore consider a more limited range around the resting or flying default values, with environmental parameters removed (table 3). In this case the parameter E also becomes important. In the limited case, the parameter α_{np}, which is the fraction of the surface area exposed to non-point sources of radiation, is also influential. However, we have no reason to believe that this parameter naturally varies, and so we disregard it. The parameters M_b and v (body mass and flight speed) are both naturally varying between individuals, and individuals can fly at different speeds. Therefore, we use these two parameters to explore the natural variation in the thermal limits for sustained flight. We use the remaining parameters, i_0 and E, to fit the model to data from the literature on key air and thorax temperatures for flight and cooling behaviours.

Parameter	Bumblebee, Cooling On	Bumblebee, Cooling Off	Honeybee, Cooling On	Honeybee, Cooling Off
T_{air}	55.4	55.5	46.4	51.7
v	17.0	17.0	53.1	27.7
i_0	12.8	12.8	0.5	12.5
P	6.4	6.5	0	4.5
M_b	4.5	4.6	0	0.7
M_{th}	1.1	1.1	0	1.3

Table 2: The relative contribution of variability in parameter value to variablity in the model output from the global sensitivity analysis. Parameters not shown have neglible influence (≤ 1). Note that the precise values will change each time the proceedure is run, due to the stochastic nature of the boosted regression tree method.

	Bumblebee		Honeybee	
	Flying	Resting	Flying	Resting
v	39.7414	n\a	48.4109	n\a
i0	41.6857	45.7100	46.4314	0.0336
alpha_th	0.7897	24.3669	1.0746	80.4835
E	1.5151	3.9875	1.0572	0.0356
M_b	14.2283	19.0693	0.7614	0.0500

Table 3: The relative contribution of variability in parameter value to variablity in the model output from the limited sensitivity analysis (non-environmental parameters only). Parameters not shown have neglible influence (≤ 1). Resting and flying metabolic behaviours are treated separately. Note that the precise values will change each time the proceedure is run, due to the stochastic nature of the boosted regression tree method.

4 Fitting i_0 and E

The parameters i_0 and E are highly influential on the model output, and therefore are suitable candidates to use in model fitting. To fit the parameter values, we use an Approximate Bayesian Computation approach [33]. The steps are as follows:

1. Create a uniform grid over the specified ranges: $i_0 \in [0.0013, 0.015]$ with step size 0.0003 for bumblebees or $i_0 \in [0.000452, 0.0480]$ with step size 0.0001 for honeybees and $E \in [0, 0.7]$ with step size 0.002. This approximates the posterior distribution of i_0 and E.

2. At each point on the grid of i_0 and E, solve the model for the air temperature at which a resting bee reaches the necessary thorax temperature for flight and the air temperature at which a flying bee with no cooling via the abdomen or tongue-lashing reaches the critical thermal limit. These thorax temperatures are indicated in table 7.

3. Repeat 1000 times to generate the posterior distribution for the fitted values of i_0 and E (shown in figures 5 and 5):

   (a) Sample from the two target air temperature ranges using the Uniform distribution. These ranges are given in table 7.

   (b) Calculate the Euclidean distance of each grid point from the sample values: D = sqrt((flight target value-flight model value)^2 + cooling target value - cooling model value)^2)

   (c) Select the point with the minimum Euclidean distance and save the values of i_0 and E.

4. Using the function *kd2e* (R package Akima [1]), perform an interpolation and select the values of i_0 and E corresponding to the highest density.

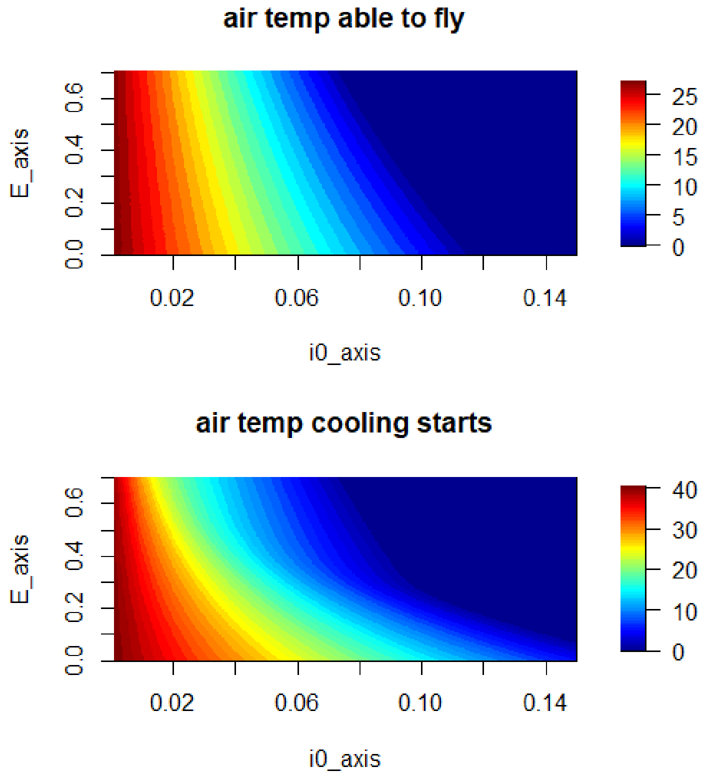

Figure 3: The air temperature (in degrees C) at which the modelled bumblebee is able to fly and the air temperature at which cooling behaviour is necessary, over the grid of i_0 and E values. Air temperatures are determined by finding the air temperature at which the model produces equilibrium thorax temperatures closest to the thorax temperatures provided in table 7.

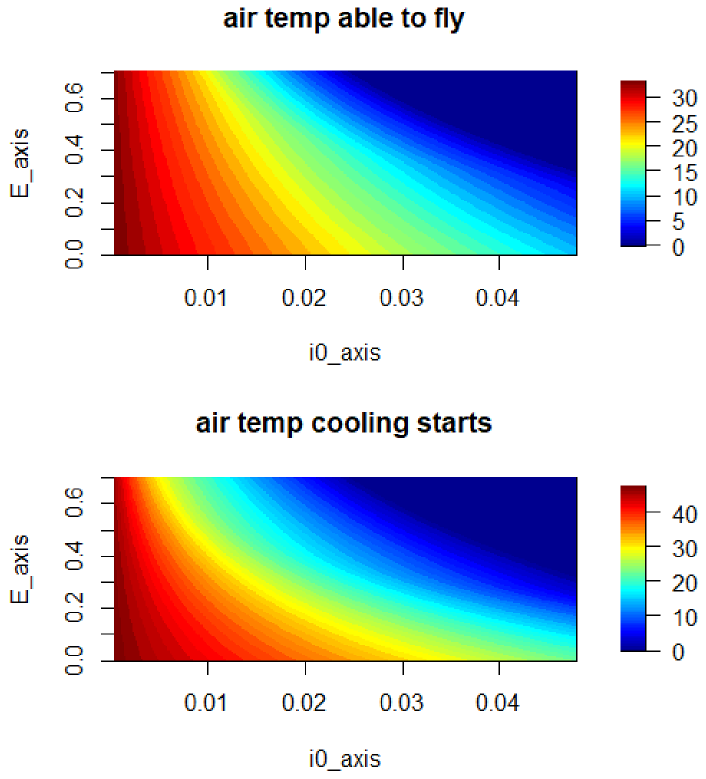

Figure 4: The air temperature (in degrees C) at which the modelled honeybee is able to fly and the air temperature at which cooling behaviour is necessary, over the grid of i_0 and E values. Air temperatures are determined by finding the air temperature at which the model produces equilibrium thorax temperatures closest to the thorax temperatures provided in table 7.

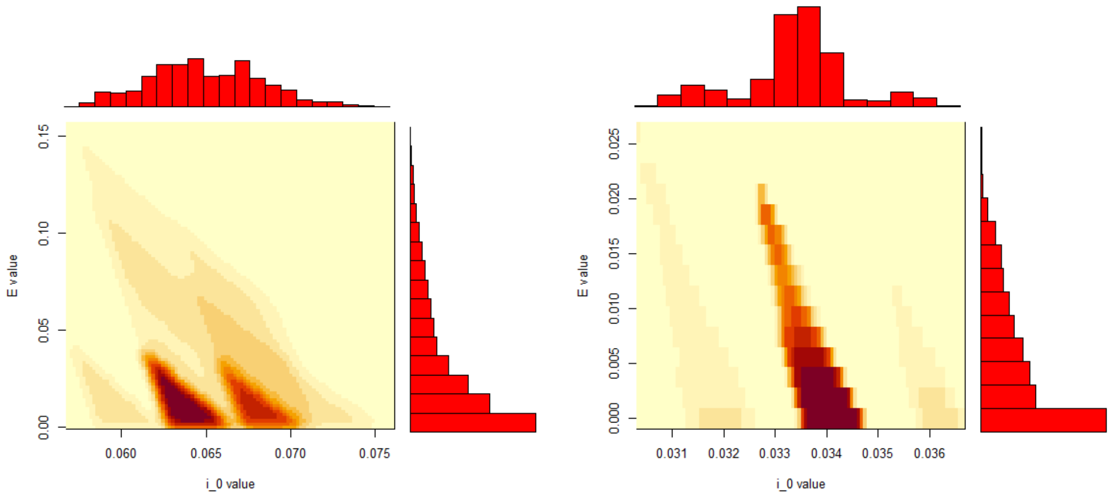

Figure 5: The interpolated density of i_0 and E values (centre panel) and histograms of the posterior distribution of i_0 (top) and E (side) for bumblebees (left) and honeybees (right). The mirrored images in the interpolated density heatmap are caused by the nonlinearity in the way air temperature for flight and cooling behaviour respond to the parameter values, and the interaction between these two air temperatures in determining the euclidean distance to the target values.

5 Parameter Values and Ranges

Description	Symbol	Default Value	Range	Source	Species
difference between thorax and head temperature*	ΔT_h	2.9°C	[1.6, 4.2]°C	[10]	*Apis mellifera*
resting metabolic rate	i_0	$1.34973 \cdot 10^{-3}$ J/s	[0.00011, 0.00824] J/s	[16]	*B. vosnesenskii*
active metabolic rate - literature	i_0	$6.229515 \cdot 10^{-2}$ J/s	[0.058424, 0.066167] J/s	[16]	*B. vosnesenskii*
active metabolic rate - fitted	i_0	$6.334973 \cdot 10^{-2}$ J/s	n/a		
reference mass for i_0	m_i	0.177 g	n/a	[16]	*B. vosnesenskii*
reference temperature for i_0	T_i	25°C	n/a	[16]	*B. vosnesenskii*
average body mass	m_b	0.149 g	[0.035, 0.351] g	[15]	
activation energy - literature	E	$0.63 \cdot 1.60210^{-19}$ J	$[0.60, 0.70] \cdot 1.60210^{-19}$ J	[2]	
activation energy - fitted	E	$0.01267 \cdot 1.60210^{-19}$ J	n/a		
average thorax mass	m_{th}	0.057 g	[0.014, 0.132] g	[15]	*B. bimaculatus, fervidus, vagans*
specific heat capacity of insect tissue	c	$3.349 J \cdot g^{-1} \cdot K^{-1}$	±10%	[20]	
rate of heat transfer to abdomen	r_0	0.004 J/s/°C	[0.002, 0.008] J/s/°C	[9]	*B. vosnesenskii*
median thorax temperature at which abdomen cooling begins	T_c	42°C	[40, 44]°C	[9]	*B. vosnesenskii*
fraction of surface area irradiated (shape factor) by point sources	α_p	0.25	±10%	[23]	
absorptivity	ϵ_a	0.935	[0.920, 0.950]	[34]	*B. terrestris, pascuorum*
surface area of the thorax	a_{th}	$9.3896 \cdot 10^{-5}$ m^2	$[8.8247, 10.5683] \cdot 10^{-5}$ m^2	calculated from d_{th}	
surface area of the head*	a_h	$3.61 \cdot 10^{-5}$ m^2	$[3.02, 4.50] \cdot 10^{-5}$ m^2	personal data	*B. terrestris*
fraction of surface area irradiated by non-point sources	α_{np}	0.5	±10%	[7, 23]	

emissivity*	ϵ_e	0.970	[0.955, 0.990]	[31]	*Apis mellifera carnica*
convection heat transfer coefficient	h	function of T_{air}	n/a	[5, 21]	
surface to internal temperature ratio	s	0.9965	[0.99, 1.00]	[5]	*B. lapidarius*
convection scaling parameter	c_d	$2.4 \cdot 10^{-7}$	$[2.3, 2.6] \cdot 10^{-7}$	[5, 21]	
convection exponential parameter	n	1.975485	[1.967937, 1.983033]	[5, 21]	
averge thorax diameter	d_{th}	0.005467 m	[0.005300, 0.005800] m	[5]	*B. terrestris, lapidarius, derhamellus*
flight speed	v	4.1 m/s	[1.0, 5.5] m/s	[25]	*B. terrestris*
initial thorax temperature	T_0	30°C	[20, 39]°C	[8, 18]	*B. impatiens,* unspecified
critical thermal maximum	CT_{max}	42°C	n/a	[26]	*B. impatiens*
minimum thorax temp for flight	T_{min}	30		[11]	*B. terricola*
thorax temperature at which abdomen cooling begins	T_c	42°C	[40, 44]°C	[9]	*B. vosnesenskii*

Table 4: Parameter values in the heat exchange equations for bumblebees, presented in order of first appearance in this manuscript. In a few cases (marked *), when values for bumblebees were not available we used honeybee values. The ranges provided for each parameter are used in the sensitivity analysis. When a range was not avalable from the source paper, we used a default range of ±10%, commonly used in sensitivity analyses.

**Description**	**Symbol**	**Default Value**	**Range**	**Source**	**Species**
difference between thorax and head temperature	ΔT_h	2.9°C	[1.6, 4.2]°C	[10]	*Apis mellifera*
resting metabolic rate	i_0	$4.52 \cdot 10^{-4}$ J/s	±10%	[24, 29]	*Apis mellifera*
active metabolic rate - literature	i_0	$3.20 \cdot 10^{-2}$ J/s	[4.52 , 4.80] $\cdot 10^{-2}$J/s	[24, 29]	*Apis mellifera*
active metabolic rate - fitted	i_0	$3.3452 \cdot 10^{-2}$ J/s	n/a		
reference mass for i_0	m_i	80 g	n/a	[24, 29]	*Apis mellifera*

reference temperature for i_0	T_i	25°C	n/a	[24, 29]	
average body mass	m_b	0.1008 g	SD = 0.0202 g	[7]	*Apis mellifera*
activation energy - literature	E	$0.63 \cdot 1.60210^{-19}$ J	$[0.60, 0.70] \cdot 1.60210^{-19}$ J	[2]	not specific to bees
activation energy - fitted	E	$0.008 \cdot 1.60210^{-19}$ J	n/a		
average thorax mass	m_{th}	0.0407 g	SD = 0.0029 g	[7]	*Apis mellifera*
specific heat capacity of insect tissue	c	$3.349 J \cdot g^{-1} \cdot K^{-1}$	±10%	[20]	
median head temperature at which evaporative cooling begins	T_c	45°C	[44, 46]°C	[10, 12]	*Apis mellifera*
fraction of surface area irradiated (shape factor) by point sources	α_p	0.25	±10%	[23]	
absorptivity	ϵ_a	0.91	0.90-0.92	[34]	*Apis mellifera*
surface area of the thorax	a_{th}	$4.50 \cdot 10^{-5}$ m^2	SD = $0.29 \cdot 10^{-5}$	[7]	*Apis mellifera*
surface area of the head	a_h	$2.46 \cdot 10^{-5}$ m^2	SD = $0.43 \cdot 10^{-5}$	[7]	*Apis mellifera*
fraction of surface area irradiated by non-point sources	α_{np}	0.5	±10%	[7, 23]	
emissivity	ϵ_e	0.970	[0.955, 0.990]	[31]	*Apis mellifera carnica*
convection heat transfer coefficient	h	function of T_{air}	n/a	[5, 21]	
surface to internal temperature ratio*	s	0.997	[0.990, 1.000]	[5]	*B. lapidarius*
convection scaling parameter*	c_d	$2.4 \cdot 10^{-7}$	$[2.3, 2.6] \cdot 10^{-7}$	[5, 21]	
convection exponential parameter*	n	1.975485	[1.967937, 1.983033]	[5, 21]	
averge thorax diameter	d_{th}	0.004 m	±10%	[5, 22, 34]	*Apis mellifera*
flight speed	v	5.6 m/s	SD 1.0 m/s	[3]	*Apis mellifera*
initial thorax temperature	T_0	39°C		[7]	*Apis mellifera*
radius of nectar drop	R_0	$3.08 \cdot 10^{-4}$ m	$[1.20 \cdot 10^{-4}, 4.50 \cdot 10^{-5}]$	personal data	*Apis mellifera*
critical thermal maximum	CT$_{max}$	52°C	n/a	[6, 13, 19]	*Apis mellifera*

minimum thorax temp for flight	T_{min}	35°C	n/a	[12]	*Apis mellifera*
head temperature at which evaporative cooling begins	T_c	45°C	[44, 46]°C	[10, 12]	*Apis mellifera*

Table 5: Parameter values in the heat exchange equations for honeybees, presented in order of first appearance in this manuscript. In a few cases (marked *), when values for honeybees were not available we used bumblebee values. The ranges provided for each parameter are used in the sensitivity analysis (see Supporting Information for details). When a range was not avalable from the source paper, we used a default range of ±10%, commonly used in sensitivity analyses.

Description	Symbol	Default Value	Range	Source
Boltzmann constant	k	$1.3806 \cdot 10^{-23}$ J/K	n/a	
ground reflectance	f	0.25	[0.17, 0.32]	[7, 27]
solar radiation	P	332.39 W m^{-2}	[29.87, 1041.00] W m^{-2}	typical for Ireland
Stefan–Boltzmann constant	σ	$5.67 \cdot 10^{-8}\ W \cdot m^{-2} \cdot K^{-4}$	n/a	
ground surface temperature	T_g	17.1°C	[3.1,19.6]°C	typical for Ireland
empirically determined parameter	δ	$5.31 \cdot 10^{-13}$	n/a	[32]
air temperature	T_{air}	n/a	[0, 50]°C	
thermal conductivity of air	κ	function of T_{air}	n/a	
kinematic viscosity of air	ν	function of T_{air}	n/a	
latent heat of vaporisation of water	h_{fg}	$2.3819 \cdot 10^{6}$ J/kg	±10%	[35]
relative humidity	rh	0.6908	[0.3920, 0.9349]	[17]
molar mass of dry air	MM_{air}	0.0289652 kg/mol	n/a	
molar mass of water vapor	MM_{vapor}	0.018016 kg/mol	n/a	
Clausius-Clapeyron constant for water	A	$9.1496 \cdot 10^{10}$	n/a	[30]
Clausius-Clapeyron constant for water	B	$-5.1152 \cdot 10^{3}$	n/a	[30]
Atmospheric pressure	Pr	$1.01325 \cdot 10^{5}$ N/m^2	n/a	
specific gas constant for dry air	$R_{specific}$	287.058 J/kg/K	n/a	

Table 6: Physical constants, environmental parameters, and sensitivity analysis ranges where applicable. Physical constants are not varied. These parameters are all the same for both bumblebees and honeybees.

	**Bumblebee**			**Honeybee**		
	**Thorax**	**Air**	**Source**	**Thorax**	**Air**	**Source**
Minimum for flight	30°C	9-11 °C	[11]	34-36 °C	9-11 °C	[12]
Cooling behaviour begins	42°C	20-25°C	[9]	47.9°C	35°C	[10, 12]

Table 7: Key air and corresponding thorax temperatures used to fit i_0 and E.

References

[1] Akima, Hiroshi, & Gebhardt, Albrecht. 2022. *akima: Interpolation of Irregularly and Regularly Spaced Data*. R package version 0.6-3.4.

[2] Brown, James H., Gillooly, James F., Allen, Andrew P., Savage, Van M., & West, Geoffrey B. 2004. Toward a Metabolic Theory of Ecology. *Ecology*, **85**(7), 1771–1789.

[3] Capaldi, Elizabeth A, Smith, Alan D, Osborne, Juliet L, Fahrbach, Susan E, Farris, Sarah M, Reynolds, Donald R, Edwards, Ann S, Martin, Andrew, Robinson, Gene E, Poppy, Guy M, & Riley, Joseph R. 2000. Ontogeny of orientation flight in the honeybee revealed by harmonic radar. *Nature*, **403**, 537–540.

[4] Carnell, Rob. 2020. *lhs: Latin Hypercube Samples*. R package version 1.0.2.

[5] Church, Norman Stanley. 1960. Heat Loss and the Body Temperatures of Flying Insects: II. Heat conduction within the body and its loss by radiation and convection. *Journal of Experimental Biology*, **37**, 166–212.

[6] Coelho, Joseph R. 1991. Heat Transfer and Body Temperature in Honey Bee (Hymenoptera: Apidae) Drones and Workers. *Environmental Entomology*, **20**(6), 1627–1635.

[7] Cooper, Paul D., & Schaffer, William M. 1985. Temperature Regulation of honey bees (*Apis mellifera*) foraging in the sonoran desert. *Journal of Experimental Biology*, **114**, 1–15.

[8] Heinrich, Bernd. 1974. Thermoregulation in Bumblebees I. Brood Incubation by *Bombus vosnesenskii* Queens. *Journal of comparative physiology*, **88**, 129–140.

[9] Heinrich, Bernd. 1976. HEAT EXCHANGE IN RELATION TO BLOOD FLOW BETWEEN THORAX AND ABDOMEN IN BUMBLEBEES. *Journal of Experimental Biology*, **64**, 561–585.

[10] Heinrich, Bernd. 1980. MECHANISMS OF BODY-TEMPERATURE REGULATION IN HONEY-BEES, *Apis mellifera* I. REGULATION OF HEAD TEMPERATURE. *Journal of experimental Biology*, **85**, 61–72.

[11] Heinrich, Bernd. 1983. Do Bumblebees Forage Optimally, and Does it Matter? *American Zoology*, **23**, 273–281.

[12] Heinrich, Bernd. 1987. *Neurobiology and Behavior of Honeybees*. Springer Verlag. Chap. Thermoregulation by Individual Honeybees, pages 102–111.

[13] Helmut Käfer, Helmut Kovac, Anton Stabentheiner. 2012. Resting metabolism and critical thermal maxima of vespine wasps (*Vespula* sp.). *Journal of Insect Physiology*, **58**, 679–689.

[14] Hijmans, Robert J., Phillips, Steven, Leathwick, John, & Elith, Jane. 2017. *dismo: Species Distribution Modeling*. R package version 1.1-4.

[15] Joos, B., Young, P. A., & Casey, T. M. 1991. Wingstroke frequency of foraging and hovering bumblebees in relation to morphology and temperature. *Physiological Entomology*, **16**, 191–200.

[16] Kammer, Ann E., & Heinrich, Bernd. 1974. Metabolic rates related to muscle activity in bumblebees. *Journal of Experimental Biology*, **61**, 219–227.

[17] Karbassioon, A., Yearlsey, J., Dirilgen, T., Hodge, S., Stout, J. C., & Stanley, D. A. 2022. *Responses in honeybee and bumblebee activity to changes in weather conditions*. Manuscript submitted for publication.

[18] Kelemen, Evan, & Dornhaus, Anna. 2018. Lower temperatures decrease worker size variation but do not affect fine-grained thermoregulation in bumble bees. *Behavioral Ecology and Sociobiology*, **72**(170).

[19] Kovac, Helmut, Käfer, Helmut, Stabentheiner, Anton, & Costa, Cecilia. 2014. Metabolism and upper thermal limits of *Apis mellifera carnica* and *A. m. ligustica*. *Apidologie*, **45**, 664–677.

[20] Krogh, August, & Zeuthen, Erik. 1941. THE MECHANISM OF FLIGHT PREPARATION IN SOME INSECTS. *Journal of Experimental Biology*, **18**(1), 1–10.

[21] Mills, A. F., & Coimbra, C. F. M. 2016. *Heat Transfer*. 3 edn. Temporal Publishing, LLC.

[22] Mitchell, John W. 1976. Heat transfer from spheres and other animal forms. *Biophysical Journal*, **16**(6), 561–569.

[23] Montieth, John L., & Unsworth, Mike H. 2013. *Principles of Environmental Physics: Plants, Animals, and the Atmosphere*. 4 edn. Academic Press.

[24] Nachtigall, W., Rothe, U., Feller, Paulette, & Jungmann, R. 1989. Flight of the honey bee III. Flight metabolic power calculated from gas analysis, thermoregulation and fuel consumption. *Journal of Comparative Physiology B*, **158**, 729–737.

[25] Osborne, Juliet L., Smith, Alan, Clark, Suzanne J., Reynolds, Don R., Barron, Mandy C., Lim, Ka S., & Reynolds, Andy M. 2013. The Ontogeny of Bumblebee Flight Trajectories: From Naïve Explorers to Experienced Foragers. *PLoS ONE*, **8**(11).

[26] Oyen, K. Jeannet, & Dillon, Michael E. 2018. Critical thermal limits of bumblebees (*Bombus impatiens*) are marked by stereotypical behaviors and are unchanged by acclimation, age or feeding status. *Journal of Experimental Biology*, **221**(8).

[27] Page, J K. 1979. *Solar Energy Conversion: An Introductory Course*. Pergamon. Chap. METHODS FOR THE ESTIMATION OF SOLAR ENERGY ON VERTICAL AND INCLINED SURFACES, pages 37–99.

[28] Prowse, Thomas A. A., Bradshaw, Corey J. A., Delean, Steven, Cassey, Phillip, Lacy, Robert C., Wells, Konstans, Aiello-Lammens, Matthew E., Akçakaya, H. R., & Brook, Barry W. 2016. An efficient protocol for the global sensitivity analysis of stochastic ecological models. *Ecosphere*, **7**(3), e01238. 10.1002/ecs2.1238.

[29] Rothe, U., & Nachtigall, W. 1989. Flight of the honey bee IV. Respiratory quotients and metabolic rates during sitting, walking and flying. *Journal of Comparative Physiology B*, **158**, 739–749.

[30] Sidebotham, G. 2015. *Heat Transfer Modeling*. Springer, Cham. Chap. Evaporation and Mass Transfer Fundamentals.

[31] Stabentheiner, Anton, & Schmaranzer, Sigurd. 1987. Thermographic Determination of Body Temperatures in Honey Bees and Hornets: Calibration and Applications. *Thermology*, **2**(4), 563–572.

[32] Swinbank, W. C. 1963. Long wave radiation from clear skies. *Quarterly Journal of the Royal Meteorological Society*, **89**, 339–348.

[33] Toni, Tina, Welch, David, Strelkowa, Natalja, Ipsen, Andreas, & Stumpf, Michael P H. 2009. Approximate Bayesian computation scheme for parameter inference and model selectionin dynamical systems. *Journal of the Royal Society Interface*, **6**, 187–202.

[34] Willmer, P. G., & Unwin, D. M. 1981. Field analyses of insect heat budgets: reflectance, size and heating rates. *Oecologia*, **50**, 250–255.

[35] Young, Hugh D, Freedman, Roger A, Ford, A. Lewis, Sears, Francis Weston, & Zemansky, Mark Waldo. 2016. *Sears and Zemansky's university physics with modern physics*. Fourteenth edn. Pearson Education Limited.
