# Supplementary material for: Thermal limits of bumblebees and honeybees are modulated by different functional traits: predictions of a mechanistic model
Source: PLoS One. 2025 May 6;20(5):e0320038. doi: 10.1371/journal.pone.0320038 (PMC12054886; doi:10.1371/journal.pone.0320038)
Supplement: Attachment — Submitted filename: ResponsesToReviewers_2024-10-10.odt [file pone.0320038.s002.odt]

Reviewers' comments:

Reviewer's Responses to Questions
Comments to the Author

1. Is the manuscript technically sound, and do the data support the conclusions?

The manuscript must describe a technically sound piece of scientific research with data that supports the conclusions. Experiments must have been conducted rigorously, with appropriate controls, replication, and sample sizes. The conclusions must be drawn appropriately based on the data presented.
Reviewer #1: Yes
Reviewer #2: Partly

2. Has the statistical analysis been performed appropriately and rigorously?
Reviewer #1: Yes
Reviewer #2: N/A

3. Have the authors made all data underlying the findings in their manuscript fully available?

The PLOS Data policy requires authors to make all data underlying the findings described in their manuscript fully available without restriction, with rare exception (please refer to the Data Availability Statement in the manuscript PDF file). The data should be provided as part of the manuscript or its supporting information, or deposited to a public repository. For example, in addition to summary statistics, the data points behind means, medians and variance measures should be available. If there are restrictions on publicly sharing data—e.g. participant privacy or use of data from a third party—those must be specified.
Reviewer #1: Yes
Reviewer #2: No

Note from the authors: We have added a brief section at the end of the paper with information on where to find the code and data, which is publicly available via an OSF repository at https://osf.io/sajkt/. 

4. Is the manuscript presented in an intelligible fashion and written in standard English?

PLOS ONE does not copyedit accepted manuscripts, so the language in submitted articles must be clear, correct, and unambiguous. Any typographical or grammatical errors should be corrected at revision, so please note any specific errors here.
Reviewer #1: Yes
Reviewer #2: Yes

5. Review Comments to the Author

Please use the space provided to explain your answers to the questions above. You may also include additional comments for the author, including concerns about dual publication, research ethics, or publication ethics. (Please upload your review as an attachment if it exceeds 20,000 characters)


Reviewer #1: In this manuscript, the authors present a thermodynamic model for insect thorax temperatures that includes metabolic processes and behavioural adaptations. The study is interesting, thorough and paves the way for species specific models on adaptation to changing climatic conditions while considering specific behavioural adaptations. I found the manuscript easy to read and understand for the most part but have some major and minor comments:

Major comments:

1. Questions on Qh: I found the explanation of how Qh is calculated difficult to understand. From Fig. 1, Qh is implied as heat taken away from thorax. In Eq. 1, Qh is added to (and not subtracted from) the thoracic temperature. In the text (L104-109) Qh is a combination of heat gain and loss due to environmental processes similar to what is described for the thorax. However, for honey bees the authors point out that the temperature differential between head and thorax is maintained at a constant value irrespective of this heat gain and loss. I think the authors should improve their description of Qh to make it’s definition and role clearer.
Because Q_H itself is calculated by adding environmental heat gain and subtracting environmental heat loss, it will be negative when the head is losing heat. Thus, we have to add it to the model in order for it to have the effect of subtracting heat. Heat transfer from the thorax to the head was found to be primarily due to passive conduction (Heinrich 1980a), so maintaining T_h = T_th- ΔT_h effectively models this passive heat transfer from the thorax to the head. As the head cools due to environmental heat transfer, it draws more heat from the thorax to maintain that temperature differential, cooling the thorax as well.
We have designed the model in this way to avoid having to either solve for T_h or to model the rate of heat transfer from the thorax to the head, both of which would require additional assumptions (and/or empirical data that we are lacking) and increase the complexity of the model and analysis. We have added text clarifying these points when the model is introduced (lines 70-73) and in the section on physiological heat flux (lines 131-136).


2. Several assumptions of parameter values have been made in the model without clear explanations. For example, one assumption in the model related to Qh is that the temperature differential between head and thorax is the same for bumblebees as it is for honey bees. This assumption is not present in the main text but is marked in the supplementary table 4. This and other assumptions should be explicitly stated and justified in the Methods section and should not be delegated only to the supplementary information as it is essential to understand the model.
Thanks for pointing this out. We made the assumption about the differential between head and thorax temperature for bumblebees lacking any empirical evidence one way or the other. (See response to point 1 for further justification.) We have also clarified that in general, whenever information about either honeybees or bumblebees is lacking, we use the information we have for the other bee group. 

3. Discussion regarding the effect of body mass: I found some of the inferences and discussion on this topic circular. In the model inputs, the body mass of bumblebees and honey bees are input with different levels of variation (for bumblebees the range is nearly 10 fold while the variation in honey bees is much less). While I understand that this corresponds to the extant variation seen, this makes it difficult to disentangle whether the effects of body mass on flight in the model is simply due to the reduced variation. One way would be to model similar levels of variation around the mean body mass in honey bees and bumblebees. While artificial, this will help strengthen the authors’ claim of body mass being an important factor for bumblebees but not honey bees.
We addressed this in Figure 3, where we simulated both bumblebees and honeybees across a range of thorax masses from 0.01g to 0.16g. This range is wider than the realistic range for both honeybees and bumblebees. The patterns we describe are observed across this entire (unrealistic) range. We have added text to the results (lines 236-247) and discussion (lines 346-350) to clarify this point. 

4. Behavioural cooling mechanisms: This was an interesting addition to the model. However, I have some questions. Why is active cooling through evaporation of nectar only possible during flight in honey bees? The discussion states that it is strongest during flight (L220) but in the model it is taken to be completely absent (and not weaker) during rest. Can the authors clarify why they chose to not include it as a behavioural process in resting honey bees?
Any movement of air over the bee would give rise to evaporative cooling, so the underlying structure of the model allows for evaporative cooling when the bee isn’t flying. However, to simplify the model, we decided to keep wind speed to zero. Adding in wind would complicate the interpretation of the results, as we would have to include multiple wind speeds, and decide how many and what wind speeds to consider, as well as any directional effects while the bee is flying.
We have added text to clarify these points in the methods (lines 162-164) section, and modified the discussion (lines 315-319) to reflect the possible effects of wind during resting behaviour. 

5. Fig.2: I think this figure could be expanded by adding panels from Supplementary figure 1. The results on temperature ranges for flight with and without cooling are an important part of the manuscript (it is highlighted in the first paragraph of the discussion, L189-194) and it is not clear why they are not presented in the main results.
We originally thought that figure 2 (now figure 3) as presented in the original manuscript gave a clearer picture, but upon reflection we agree with the reviewer that the figure as presented in the supplementary material is better. We have updated the text in the discussion (lines 263-272) to better reflect the new figure.
Although we believe the reviewer was referring to Supplementary figure 2 here (which corresponds to what was originally figure 2 in the main manuscript) we think that  Supplementary figure 1 also enhances the main manuscript, and have therefore included it To accommodate this, we added a short section to the methods (Model Simulations, lines 193-196), a description of the results at the beginning of the results section (lines 198-201), and a new figure, now figure 2.


6. L143-145: I had difficulty understanding how and why the parameter E was chosen for the restricted sensitivity analysis, given that it only explains very little amount of the variation (supplementary tables 3 and 4). E is only present when non-environmental variables are considered and even then, it only accounts for 1-3% of the remaining variation. The authors should justify their choice of using E more clearly.
Thank you for pointing out this lack of clarity. When we ran the model with the values for E suggested by the literature (Brown et. al, 2004), it produced unrealistic results with respect to the expected temperature thresholds for flight. The limited sensitivity analysis (keeping environmental variables constant) revealed that the metabolic rate i_0, the flight speed, v, the mass of the bee m_b, and the activation energy E are the top four variables accounting for the variability in the model results. Of these four parameters, E and i_0 are fixed physiological characteristics,  v is a behaviour that can vary, and m_b is a naturally varying trait. We therefore fit i_0 and E to achieve as close to the expected temperature thresholds as possible, and explore the model results with respect to varying v and m_b. We have updated the methods section (lines 175-178) with this information. 

7. In the parameters which require input of surface area values, only the surface area of the thorax or the head is provided in the various equations. Is heat gain or loss through the abdomen negligible? Can the authors elaborate on this.
Due to the very narrow connection between the thorax and abdomen, heat loss from the thorax to the abdomen is negligible, and we therefore disregard the abdomen except in the case of behavioural cooling by bumblebees. When behavioural cooling using the abdomen is activated, we simplify the model by considering the abdomen to be a heat sink and its temperature to be the same as the ambient air temperature. That is, heat flows from the thorax to the abdomen at a given rate, and then is disregarded..
To clarify this point, we have moved the text explaining the counter-current heat exchange from the Behavioural Mechanisms subsection to the Physiological Mechanisms subsection (lines 137-140) and updated the description of Q_A (lines 142-148). We have also added some text to the beginning of the methods section explaining that we are only directly modelling the thorax temperature, and how the head and abdomen factor into this (lines 50-55). 

Minor comments:

8. The introduction was well written and covered the major points succinctly. I enjoyed reading this section.
Thank you!

9. L64: It is not clear to me why certain subscripts in Eq 1 are in small letters (Qab) compared to others? What is the convention being used?
The convention being used is human error. We have no idea why “ab” and “n” were in lower case. We have changed Q_ab to Q_A to be consistent with the other one letter capital subscripts and Q_n to Q_N for consistency with the capitalization. 

10. L96: How is the average thorax diameter calculated?
We took the average value from a few different sources in the literature. We have added text to this effect in the manuscript (Lines 108-109). 

11. L108: the section number is missing.
Fixed

12. L210: Bumblebees is wrongly spelt as 'bumblebeest'
Oops, thanks!


13. L229-230: I’m not sure how cooling capacity benefits bumblebees more mid-foraging given that in both species the active cooling process is present during flight.
Cooling capacity could benefit bumblebees mid-foraging because they could stop to rest and keep cooling via the abdomen while resting, unlike honeybees. We have updated the text to include this information (lines 336-337). In the course of responding to reviewer 2’s comments we have also shifted this discussion away from bumblebees vs. honeybees and focussed on the types of cooling mechanism. 

14. L262 ‘improce’ should be replaced with ‘improve’.
Oops, thanks!


Reviewer #2: This paper presents a mechanistic model for predicting the thermal limits of activity in honeybees and bumblebees to facilitate predictions of how future climate changes will affect crop pollination. The model is based primarily on empirical data but is designed to be applied beyond these limits. In general, the text is well-written but is lacking important clarifications and includes generalisations and implicit assumptions that make the results of the model difficult to interpret and would make it problematic for other research groups to apply. As such, the work is not suitable for publication in its present form but could be if the concerns below are adequately addressed.

General comments
While the authors’ attempt to develop a general model by including two groups of bees is commendable, the work is limited by the lack of empirical data from more than a few species (and only one for honeybees). The general oversight regarding potential differences between honeybee and bumblebee species that inhabit different environments is the greatest weakness of the work. Although it is understandable that the authors are necessarily limited because the empirically measured parameters required for the model are only available for a limited number of species, it is important for this limitation to be made explicit. For example, bumblebee species (of which there are approximately 250) occupy a broad range of habitats, including the Arctic, the Mediterranean, the deserts of North America and the Amazon rainforest. Honeybee species also vary in both their habitat and their body size, which would also potentially affect the generalisability of the results. Because of this, it is unlikely that the parameters used for the model apply to species that have evolved in these more extreme environments and it is therefore important that the authors are aware of this and discuss this limitation in the text. The other problem associated with the limited number of species used in the model is that the conclusion that honeybee body size does not affect their thermal limits may be incorrect. This is because the honeybee species from which all empirical data was made tends to have workers that do not vary in body size. However, different honeybee species have very different body sizes and this could result in differences in thermal limits. As the authors do not clarify that the honeybee species they refer to is Apis mellifera, this leads to the impression that these results would apply to other honeybee species with different body sizes, but this may not be the case.

Thank you for this thoughtful feedback. We have made changes throughout the text to clarify that we are referring to Apis mellifera, as well as bumblebees specifically from temperate regions. We have also added a paragraph to the discussion about the limitations of using only a few species to parameterise our model. (Lines 383-392)

Line 31, 55 and line 181: the model apparently only accounts for temperature changes and not changes in other weather factors, such as cloud cover, wind or humidity. If this is not the case, then please specify what other weather variables were used, if this is the case then clarify by replacing ‘weather’ here with ’temperature’.
Thank you for pointing this out. Temperature is an important weather variable, but it is not the only weather variable used in the model. The model includes solar radiation, ground surface temperature, air temperature, and relative humidity. We have updated the manuscript to reflect this in lines 44-45 and modified the methods section to include this information and define the weather variables where appropriate. The model variables are all defined in Table 6 of the supporting materials.


Equation 1: define the terms dt and dTth in the text before presenting the model
We have updated the text (lines 56-57) to include this information

Equation 1: please also explain why QH is being added rather than subtracted here. Potential heat transfer from the thorax to the head would reduce the thorax temperature rather than increase it as suggested in this equation. Please clarify the reasoning behind this in the text.
We have added text clarifying this reasoning when the model is introduced (lines 70-73) and in the section on physiological heat flux (lines 131-136).  

Line 68: This statement should be supported with an explanation or a citation “and head transfer from the thorax to the head are necessary for bees”. There appears to be only two published studies that explore heat transfer to the head in bees and it appears as though they can regulate this. These works should be cited and the statement revised to reflect the state of our knowledge on this:

Meredith G. Johnson, Jordan R. Glass, Jon F. Harrison; A desert bee thermoregulates with an abdominal convector during flight. J Exp Biol 1 October 2022; 225 (19): jeb244147. doi: https://doi.org/10.1242/jeb.244147

Guadalupe Sepúlveda-Rodríguez, Kevin T. Roberts, Priscila Araújo, Philipp Lehmann, Emily Baird, Bumblebee thermoregulation at increasing temperatures is affected by behavioral state, Journal of Thermal Biology, 2024, 103830,
https://doi.org/10.1016/j.jtherbio.2024.103830.

Thank you for these references! We have updated the text to “...and head transfer from the thorax to the head occur in bees” to be more accurate and included the bumblebee reference (line 66).  We added the information about bumblebees to the methods section as well as expanding on our source for heat transfer to the head in honeybees (lines 119-123). The reference on Sonoran desert bees fits well in the second to last paragraph of our discussion, so we have added it there (lines 400-402). We also added both of these references to the relevant part of the introduction (lines 30-31).

Line 74: Isn’t the reflection of shortwave radiation from the Earth dependent on the substrate (i.e. if it is long grass, short grass, mud, water etc.)? If so, please explain the assumption of reflectance being made for this equation and note any potential caveats about substrate in the text.

The reflectance is included in the model as the parameter f, as specified in line 85. Our default value if f = 0.25, which is the value for a grassy surface, but in the sensitivity analysis we did not find f to be significant. We have now included this default value in the text (line 86).

Equation 2: define αP in the text

P is defined in line 84.

Equations 2 and 3: These equations are quite confusing. Why is the surface area of the bee’s thorax specifically important for the calculations of Qs and QL and why is it treated separately from the body surface calculations? Please clarify in the text.
The surface area of the thorax is specifically important because we are modelling the bee’s thorax. Heat flux is defined per unit area, so the heat flux incident on the bee’s thorax (i.e. Q_S and Q_L) requires multiplying by the thorax surface area. References to the bee’s surface should indicate the thorax. This was an oversight on our part in the wording and has now been fixed throughout the paper.

Line 84: Where does this measure of ground temperature come from? Please explain in the text.
This was taken from Met Éireann  for typical values for Ireland (https://www.met.ie/climate/30-year-averages). This information is in the supplementary materials, but we have now included it in the text in line 92. 

Line 103: This is quite confusing. The resting metabolic rate was used for the model but then applied to flying as well but these are two very different values in most insects. Furthermore, the metabolic rate for flight varies with air temperature. Please clarify how using the resting metabolic rate value is valid.
This is a good point. Lacking any other model for the metabolic rate, we have used the same functional form of metabolic rate (with different parameter values for i_0)  for flight as well as resting. We have modified the text to explain this (lines 115-117).  There is also a discussion of the impacts of using the resting metabolic rate model for flying metabolic rate in lines 363-376 of the discussion. We have modified this discussion to clarify that it refers to Q_I.


Line 104: The basis for this assumption is unclear. Why would a connection between the head and thorax affect the heat flux from the head to the environment? Would active transfer of warm haemolymph from the thorax to the head (as appears to be the case in bumblebees) affect this assumption? If so, how?
Thanks for pointing this out. It’s actually that the heat transfer from the thorax to the head plus heat flux from the head to the environment allow the thorax to cool. We had used the term “passive” here to distinguish it from the controlled transfer of heat to the abdomen. Bumblebees sending heat to the abdomen appears to be a behaviour that can be controlled and only happens above a certain thorax temperature, whereas flow of heat to the head (via warm haemolymph) seems to happen regardless of thorax temperature. We have modified the text in the manuscript (lines 119-136) to better explain this mechanism.


Line 105: This statement about a constant difference in head temperature was based on studies from honeybees and does not appear to be true for bumblebees or desert bees. The model should be revised to reflect this
It is the nature of modelling to have to make assumptions in the absence of information. Since we don’t have a study showing the relationship between head and thorax temperature over time for a flying bumblebee, since the two bee groups have similar body structures, we have chosen to assume that the difference between head and thorax temperature is the same for honeybees and bumblebees, but it is true that this may not be the case. We have updated the text with this information (lines 123-125)

Line 108: the reference to a section (in parentheses) is incomplete.

Oops, thanks! We have fixed this.

Line 116: The paper cited here is a study on honeybees and not on bumblebees, this value would also depend on whether the bees are resting or flying. Please clarify for the reader what conditions this statement is specific for.

The decision to keep the abdomen temperature fixed at the air temperature was primarily made so as not to have to directly model the abdomen temperature. We have updated this line accordingly (line 145-147). We have also changed some of the discussion to address this issue (lines 322-331). Based on the Sepúlveda-Rodríguez paper, we cannot claim that the abdomen temperature of a flying bee is maintained at a constant difference from either the thorax temperature or the air temperature as these values vary, so we feel that assuming the abdomen temperature is the air temperature is not unreasonable.

Line 117: the conclusion that “flight speed thus plays no role in abdomen cooling” should be more clearly supported, as convective cooling will increase with flight speed and should therefore affect the abdomen cooling.
The key here is that flight speed plays no role in the modelled cooling via the abdomen, since we have simplified the model by assuming that all heat transferred from the thorax is immediately transferred from the abdomen into the air so that the abdomen remains at air temperature. If we were modelling abdomen temperature independently, then you are correct that convective cooling and flight speed would play a significant role. We have changed the term “abdomen cooling” to “cooling via the abdomen” throughout the manuscript to be clear that we are modelling cooling of the thorax via the abdomen rather than cooling of the abdomen itself. In the process of responding to other comments, we have removed this sentence.


Table 1: Please specify the species for which these values were taken as it is important for the model and for the reader to know how specific they are. Particularly for bumblebees, there are around 250 different species that inhabit a broad range of habitats and that have different body sizes and hairiness. They likely have differences in their ability to thermoregulate and in their responses to temperature. The model likely uses data from very few, if more than one, species of honeybee and bumblebee but the authors do not highlight this as a limitation. Please address this in the Table and in the text, discussing how parameters taken from one, or just a few species might skew the results of the model.

We feel that it would be unwieldy to list the species used for each parameter value in the main text, but have added it to the supporting information and refer the reader there. We have also stated explicitly at the beginning of the methods section (lines 47-50) and in the table 1 caption that the parameter values come from a mixture of species. We have added a paragraph to the discussion about the impact of the limited number of species used in parameterisation.

Line 163: It is not clear what other environmental parameters the authors are referring to. Please clarify what they are here.
Thanks. We have done so. (Lines 170-172)

Line 174: when referring to ‘…ones experiencing greater wind speeds’, I assume that the authors mean head winds or would winds from other directions also have this effect? Please clarify in the text.
Upon reflection, this parenthetical statement is misleading and we have removed this phrase. Winds from other directions would in reality have this effect, but the presented model formulation does not account for the wind direction relative to the direction of flight, 

Line 189: The term ‘indefinitely’ is used incorrectly here and is misleading. Please revise.
True, while a modelled bee can fly forever, a real one of course can’t. We have removed the word ‘indefinitely’ here. (line 262)

Line 191: This conclusion does not make sense as how could bumblebees fly at 15°C if they cannot warm up their flight muscles? It is also an irrelevant statement as bumblebees do have the ability to warm up their flight muscles and to transfer heat to the abdomen during flight.
This conclusion means that at 15°C, in our model, a resting bumblebee’s thorax temperature is warm enough for it to fly. We include it here to show that the warming behaviour gives the bees a greater range of air temperatures in which they can be active. We have modified the text to clarify this (lines 266-268). 

Line 201: correct the spelling of bumblebees
Oops, thanks! Done.

Line 205: Please provide a further clarification of why bumblebees would have a higher thermal limit.
This can be seen in the solid lines on figure 4, and indicates that convective cooling is greater in bumblebees, as this is the only place where flight speed is relevant for bumblebees. We have updated the text to refer to figure 4 and added this information. (lines 291-296)

Line 209: this statement is incorrect. Some bumblebee species do inhabit tropical environments, Bombus transversalis lives in the tropical lowlands of the Amazon rainforest, for example. Other bumblebee species live in deserts so they are not limited only to cold climates as implied here. Also, the honeybee Apis mellifera evolved in the tropical rainforests of India, so it is of course going to be adapted to flight in tropical environments. Please modify this statement to make it valid.
Thank you for pointing this out. In our focus on the temperate regions where bumblebees are the most common we didn’t consider that they do exist outside these regions. We have modified the text to reflect this reality (lines 300-302).  

Line 223: This statement is incorrect as the primary behavioural cooling mechanism is dependent on flight speed as noted above (unless the authors can provide empirical data to show otherwise). Please modify.
We again make the distinction that abdomen cooling as we have modelled it is independent of flight speed. We have therefore modified the text here (line 318-319) to clarify this point and add additional discussion around the limitations of our model in this regard, and with reference to the Sepúlveda-Rodríguez paper. We have changed the comparison to be between cooling behaviours that are effective during rest or not rather than specifically honeybees and bumblebees or abdomen cooling. (lines 336-337)

Line 232: the use of the term ‘species’ here is incorrect as the terms honeybee and bumblebee are ambiguous and could refer to genera or to specific species. Please be more explicit with the terminology and what is meant by it in the text to avoid confusion.
Thanks for pointing this out. We have replaced ‘species’ with ‘bee groups’ here to match our earlier phrasing. (line 341-342)

Line 235 (and lines 244-255): As the empirical data used for honeybees in the model comes from one species, Apis mellifera, this statement is not correct without specification. Honeybee workers are not size polymorphic as bumblebee workers are, so the empirical data is likely only taken from bees that vary little in body size. This lack of size variation for honeybees and not bumblebees could have a strong effect on the conclusions made in the model and the authors should be explicit about this in the text and revise this statement to clarify.
One of the values of a mechanistic model is that it can be used to draw inferences outside of the range of measured data, unlike a statistical model, which should not be used in this way. Therefore, even though the empirical data for honeybees is taken from bees that vary little in size, extrapolating outside of that range is a valid approach. We have updated the text (lines 346-350) to include this information.

Line 236: The information in this sentence is not supported by evolution. As mentioned above, bumblebee species do live in tropical environments and they do not have smaller body sizes than those that occupy temperate climates. The authors either need to specify what species they are referring to or to include more correct information that accounts for species differences.
We have changed the text to specify bumblebees from temperate environments throughout.

6. PLOS authors have the option to publish the peer review history of their article (what does this mean?). If published, this will include your full peer review and any attached files.


If you choose “no”, your identity will remain anonymous but your review may still be made public.


Do you want your identity to be public for this peer review? For information about this choice, including consent withdrawal, please see our Privacy Policy.
Reviewer #1: No
Reviewer #2: No
