# Supplementary material for: Thermal limits of bumblebees and honeybees are modulated by different functional traits: predictions of a mechanistic model
Source: PLoS One. 2025 May 6;20(5):e0320038. doi: 10.1371/journal.pone.0320038 (PMC12054886; doi:10.1371/journal.pone.0320038)
Supplement: Attachment — Submitted filename: Response To Reviewers.odt [file pone.0320038.s003.odt]

Comments to the Author


Reviewer #1: The authors have thoroughly responded to the previous reviews and the manuscript is now much improved. I am happy to recommend this for publication. However the authors should have a look over to ensure that minor grammatical and other issues are resolved.

A few that I noticed include:
1. L26 – 29: This sentence is too long with four commas bringing together 5 subsections. I would suggest to break down this sentence and other like this into 2 or 3 to make it easier to read.
2. L49: ‘our’ should be capitalised as it is the start of the sentence.
3. L326: Please use the author names to refer to the study at the start of the sentence instead of using the reference number as it makes it harder to read.
4. L339: ‘could be allow’ should be replaced with ‘could allow’

Response: Thank you for pointing these out. We have fixed these errors and carefully read the entire manuscript to fix any other minor grammatical issues. 


Reviewer #3: This study makes a valuable contribution to understanding the effects of temperature on bees, particularly by presenting a model to measure thorax temperature. Bumblebee thorax temperature is a critical parameter influencing their ability to fly, forage, and survive under varying environmental conditions. The model developed here is an invaluable tool for investigating how bees respond to thermal stress, offering key insights into their adaptability to climate change and other stressors. By defining these physiological limits, we can better predict the resilience of bumblebee populations amidst the increasing frequency and intensity of extreme weather events, such as heatwaves.

Main Comments:

1. Include species names in methods:
While the study includes one honeybee species and several bumblebee species, the names of these species are only mentioned in the Supplementary Material. Including these species in the Methods section would enhance the manuscript's clarity and accessibility. This change would be particularly valuable for researchers focused on the variability in thermal tolerance among bees.

Response: thank you for this comment. We hadn’t included the species names for bumblebees in the methods because different parameters are drawn from different species (as not all parameters are available for any one species, unfortunately), so the model doesn’t represent any single species but rather a sort of conglomerate bumblebee. We have now provided the list of species as a footnote (from line 50). 

2. Address high CTmax in some bumblebee species:
In L345–348, the discussion suggests that a resting bumblebee would reach lethal thorax temperatures at air temperatures of 42°C. However, some studies indicate that certain bumblebee species (including those from temperate regions) can tolerate air temperatures exceeding 45°C (CTmax) (e.g. https://doi.org/10.1242/jeb.165589; https://doi.org/10.1016/j.jtherbio.2023.103672). Incorporating these findings into the discussion would provide a more comprehensive view of thermal limits.
Response: Thank you for pointing this out. We have incorporated this information into the discussion.

3. Consider the role of nutritional status:
It may be worth addressing whether nutritional status could influence an individual bee's flight activity and ability to thermoregulate under different weather conditions, particularly high temperature. This could add a valuable dimension to the study and highlight a potential avenue for future research.

Response: This is an interesting point, and although well outside the scope of our model it is worth considering. We have added a paragraph in the discussion. 

General Feedback:

The authors have done an excellent job addressing reviewers’ concerns with appropriate revisions, thoroughly explaining the model, and discussing the results in depth. I particularly appreciated the discussion on how the model can be applied to other insect species and the thoughtful consideration of its limitations.

Minor Suggestions:

• L20–22: Include the range or minimum thorax temperature required for flight, if available. This would underscore the bees’ remarkable ability to elevate thorax temperature despite ambient conditions.
• L86: Clarify whether "ground temperature" refers specifically to soil temperature or includes other surfaces, such as impervious materials, which are present in urban areas.
• L241: Specify the approximate time required for the thorax to reach equilibrium temperature, as “after some time” is a bit vague.

Additional Minor Comments:

• L55: Capitalise “Our parameters.”
• Figure 1 caption: Correct “icoming” to “incoming.”
• L76: Change “tounge” to “tongue.”
• L245: Correct “activiating” to “activating.”
• Figure 3 caption: Correct “rigth” to “right” and revise the third sentence to: “Pink curves indicate flight, and green curves indicate resting.”
• L319–325: Clarify when referring to body mass or thorax mass.  
• L338: Specify whether “higher thermal limit” refers to upper or lower thermal limits.
• L366: Change to “It is dependent.”
• L392: Revise to: “Could allow bees.”

Thank you. We have incorporated the Minor Suggestions and Additional Minor Comments.
